# The adaptive significance of large size at birth in marine snakes

**DOI:** 10.1098/rsos.231429

**Published:** 2023-12-13

**Authors:** Richard Shine, Shai Meiri, Terri G. Shine, Gregory P. Brown, Claire Goiran

**Affiliations:** ^1^ School of Natural Sciences, Macquarie University, New South Wales 2109, Australia; ^2^ School of Zoology, Tel-Aviv University, 6997801 Tel Aviv, Israel; ^3^ The Steinhardt Museum of Natural History, Tel-Aviv University, 6997801 Tel Aviv, Israel; ^4^ LabEx Corail & ISEA, Université de la Nouvelle-Calédonie, BP R4, 98851 Nouméa cedex, New Caledonia

**Keywords:** Acrochordidae, Elapidae, Hydrophiinae, Indo-Pacific, Laticaudinae, life-history evolution

## Abstract

Evolutionary shifts from one habitat type to another can clarify selective forces that affect life-history attributes. Four lineages of snakes (acrochordids and three clades within the Elapidae) have invaded marine habitats, and all have larger offspring than do terrestrial snakes. Predation by fishes on small neonates offers a plausible selective mechanism for that shift, because ascending to breathe at the ocean surface exposes a marine snake to midwater predation whereas juvenile snakes in terrestrial habitats can remain hidden. Consistent with this hypothesis, snake-shaped models moving through a coral-reef habitat in New Caledonia attracted high rates of attack by predatory fishes, and small models (the size of neonatal terrestrial snakes) were attacked more frequently than were large models (the size of neonatal sea snakes). Vulnerability to predatory fishes may have imposed strong selection for increased offspring size in marine snakes.

## 1. Introduction

The invasion of novel habitat types provides a robust opportunity to clarify the selective forces involved in life-history evolution. That opportunity is greatest when the changes in life-history traits are consistent among multiple independent replicates: that is, similar life-history changes occur in multiple lineages of organisms. For example, warm-climate species that extend their geographic range into cold climates may consistently respond via an evolutionary shift from oviparity to viviparity (in squamate reptiles [1]) or a reduction in clutch size (in birds [2]). Likewise, species that inhabit islands often exhibit different reproductive rates than do mainland taxa [3,4]. If multiple phylogenetic lineages make similar habitat transitions, we can assess the generality and causes of life-history changes induced by novel challenges in those new habitats.

The body size of offspring at birth or hatching is a fundamental life-history trait. Theoretical models suggest that optimal offspring size depends upon the relationship between neonatal body size and fitness [5]. For example, low intraspecific competition may favour an evolutionary reduction in size at birth because competitive ability is less important for offspring survival (r-selection [6]). At the other extreme, a gape-limited predator invading a habitat lacking small prey items may be under intense selection to increase offspring size, because only the largest offspring are physically capable of ingesting the available prey [7,8].

One profound habitat shift involves invasion of the oceans by terrestrial organisms. Marine habitats differ dramatically from terrestrial habitats in abiotic factors (e.g. thermal regimes, oxygen availability, light levels, ocean currents) as well as biotic factors (types of predators, prey, competitors and pathogens) [9,10]. Nonetheless, many lineages of tetrapod vertebrates have made that shift, spawning adaptive radiations of marine mammals, birds, turtles, crocodilians and squamate reptiles [11,12]. Among reptiles, the most diverse oceanic radiations comprise three clades of elapid (front-fanged) venomous snakes. One of those lineages (sea kraits, Laticaudinae) arose about 16 million years ago from terrestrial Asian elapids [13]. The other two lineages share a more recent (less than 10-million-year) common origin from Australian terrestrial elapids, but with independent transitions to fully marine existence in the aipysurines and the *Hydrophis* group [14–16]. Sea snakes from all three elapid lineages show convergent adaptations in morphology (e.g. laterally compressed body and paddle-like tail), physiology (e.g. salt-excreting glands) and life-history traits (see below). Some of these traits also occur in a fourth lineage of snakes, the Acrochordidae, that contains three taxa which use marine habitats either exclusively or occasionally [17,18].

Total clutch or litter mass of marine snakes has been reduced relative to their terrestrial ancestors, apparently because of a conflict between abdominal distension and hydrodynamics [19]. Nonetheless, offspring are large at birth in all of the marine lineages ([20,21]; see below for statistical analysis). Why should the invasion of marine habitats have favoured an increase in offspring size? We suggest that larger size at birth has been favoured because neonatal marine snakes experience higher levels of size-selective predation than do their terrestrial relatives.

A snake's body size can affect its vulnerability to predation, with smaller snakes often attacked by a wider size range of predators in terrestrial habitats (reviewed by [22]). Also, some predators actively target smaller snakes as prey [23]. A study using snake-shaped clay models reported higher rates of predator attack to smaller models, reinforcing the importance of body size (rather than behavioural shifts associated with ontogeny) as the determinant of size-related prey vulnerability [24]. In some cases, however, predation rates on terrestrial snakes can be highest on intermediate-sized individuals [25] or on larger animals [26], and the effect of body size on rate of survival may fluctuate through time [27]. Consistent with a high risk of predation, juvenile snakes of many species spend much time inactive in well-hidden retreat-sites [28] and thus are under-represented in collections relative to their abundance [29].

Although smaller size may increase vulnerability to predation in terrestrial as well as marine snakes, ocean-dwelling species have an additional liability. Because they breathe air, sea snakes must ascend to the water surface to obtain oxygen at least once every 2 h or so [30–33]. Several times a day, then, a marine snake must leave the shelter of the substrate and move up through the water column where it is vulnerable to predatory fishes and (close to the surface) birds. Records of aquatic snakes as prey of fishes and birds support the idea that surfacing to breathe is risky [34–38].

We test this hypothesis by compiling published data on body sizes at birth in marine snakes to compare to those in terrestrial species; and by experimentally testing the hypothesis that small sea snakes are exposed to intense size-selective predation.

## 2. Methods

### 2.1. Offspring sizes in marine and terrestrial snakes

**Figure 1.**
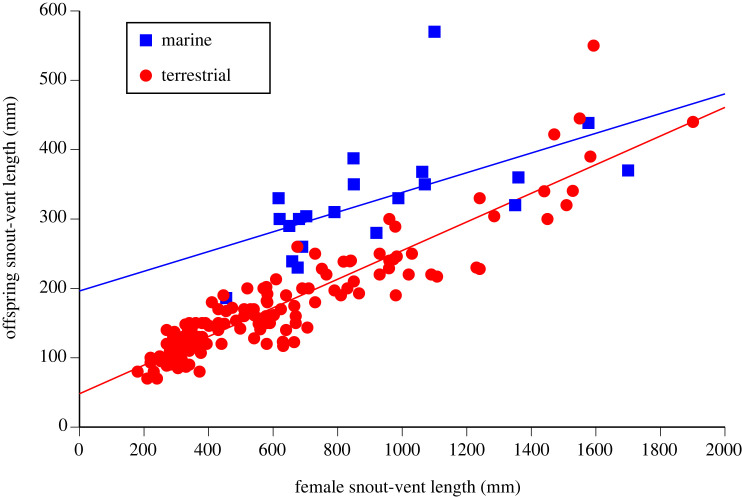
Offspring size (at birth or hatching) in marine snakes (in blue squares) compared to terrestrial (including semi-aquatic) species (in red circles). Each data point shows mean values for snout-vent length of adult females, and snout-vent length of neonates, for a single species. Regression lines are for ordinary least squares (OLS) but in the phylogenetic generalized linear (PGLS) model their slopes are not significantly different from each other (*p* = 0.13).

We obtained data on the mean snout-vent lengths (SVLs, in mm) of adult females and hatchlings (neonates in viviparous species) of 166 snake species from the collections of the Steinhardt Museum of Natural History, and from published literature on the clutch and litter sizes of these species. Sample sizes for female SVL varied among species from 1 to 1002 (mean 38, median 21; Supplementary information). Because offspring sizes of oviparous and viviparous squamates are very similar [39], we analysed viviparous and oviparous taxa together. For each species we noted whether it is marine (21 species representing all four marine snake lineages) or not (148 species). We then further classified non-marine species into semi-aquatic ones (*n* = 6) and those not associated with water (hereafter, ‘terrestrial’, though some are fossorial or arboreal). The marine *Hydrophis kingii* was a potential outlier, with a neonatal SVL of 570 mm and a mean adult female SVL of 1100 mm (figure 1).

### 2.2. Statistical analysis of life-history data

We ran phylogenetic generalized linear models (PGLS) in the R package caper [40] to compare log-transformed SVLs of neonates across marine and non-marine snakes, using log-transformed adult female SVL as an additional predictor (untransformed data resulted in qualitatively similar and with similar R^2^ values, but had much higher AIC scores; results not shown). We tested for interactions between female SVL and habitat, and found that none were significant. We used the phylogenetic tree of Tonini *et al*. [41] to quantify, and account for, phylogenetic non-independence. To this end we matched names in the tree to those we used via the reptile database (http://www.reptile-database.org/). One species missing from Tonini *et al*.'s [41] tree was omitted from our analysis. After running these analyses, we added brood size (i.e. the size of a single clutch or litter, log-transformed) as an additional covariate to test whether the potentially larger size of marine snakes is caused by them having fewer babies.

### 2.3. Experimental test of size-dependent risk of predation for small marine snakes

Average SVL of female snakes in our sample was around 800 mm (figure 1). At this size, terrestrial snakes produce offspring that are around 200 mm SVL whereas marine snakes produce offspring that are around 300 mm SVL (figure 1). Thus, the hypothesis of size-selective predation predicts higher rates of attack to a 200-mm snake than to a 300-mm one. We tested that prediction using commercially available fibreglass fishing lures (Savage Gear 3D) that are designed to resemble snakes in appearance. Each lure consists of 12 linked segments to create a sinuous movement that mimics the swimming action of a snake (see https://www.youtube.com/watch?v=PV8R3wwd9xo for video of the lure's action). Hooks were removed and replaced with lead weights to ensure negative buoyancy (see [42] for details). All lures were painted black to resemble the most common colour morph of the locally abundant sea snake *Emydocephalus annulatus* (mean SVL at birth 300 mm [42,43]). Three replicate lures of each size (200-mm or 300-mm long) were used during the trials to avoid stimulus pseudoreplication, with lure sizes changed between successive blocks of three trials per lure.

Trials were conducted during daylight hours in January 2023 on shallow-water (1 to 3 m depth) reefs beside a small island (Ile aux Canards, 22°16′S, 166°26′E) 0.8 km offshore from the city of Noumea, New Caledonia. The site is a marine reserve, where large fish are abundant and are habituated to snorkellers. The habitat, fish fauna, and local predator and snake diversity are described in another paper in which we used the larger size of these lures to test responses of predators to models of different colours [42].

For each trial, one of us (CG) pulled a lure through the water on a 5 m length of monofilament fishing line as she snorkelled in a straight line for 30 to 50 m (transect length determined by extent of consistent water-depth areas). An observer (RS) swam 3 to 5 m behind the lure to record attacks (lure seized) and follows (fish orients to lure and follows it, sometimes approaching very closely, but does not seize it). Follows as well as attacks indicate predatory intent, because all follows were by large predatory fish of species that sometimes attacked our lures (as also reported in the study by Goiran *et al*. [42]). The observer estimated body size of fishes by comparison with the length of the adjacent lure. We conducted 47 such trials.

### 2.4. Statistical analysis of experimental data

We analysed the data on outcomes per trial (whether or not an attack occurred during each trial) in SAS 9.4, using a generalized linear mixed model with negative binomial distribution and a log link function. Trial date was included as a random effect in the model because factors such as tidal stage and water clarity varied between trials conducted on different days, potentially affecting fish behaviour. Frequency distributions for fish body sizes were non-normally distributed so we classified fish as either large or small (> or <600 mm). To explore the impact of fish size on responses we used data for each fish as the unit of replication, with two independent factors (lure size and fish size, both dichotomous variables) and their interaction; fish response (follow versus attack) was the dependent variable, and day was a random variable.

## 3. Results

### 3.1. Offspring sizes in marine and terrestrial snakes

Sea snakes have larger offspring (controlling for female SVL) than do terrestrial snakes, with neonates of marine species averaging 44.2 ± 5.1% (± SE) longer than those of non-marine snakes (*t* = 7.31, *p* < 0.0001; mean values 327 mm [range 186–570, *n* = 21 for marine snake species] versus 171 mm [range 70–550 mm, *n* = 148 for non-marine ones]). Neonate SVL also increased with female SVL (slope: 0.640 ± 0.028, *t* = 23.2, *p* < 0.0001). This model explained 82.4% of interspecific variation in hatchling SVL. The phylogenetic signal was weak (*λ* = 0.346) and only marginally different from zero (*p* = 0.02). When treating semi-aquatic species separately from terrestrial ones, mean offspring sizes (corrected for female size) of semi-aquatic species were even smaller than those of marine species (marine species are 49.5 ± 9.0% larger), with hatchlings of fully terrestrial species intermediate in this respect (marine species 44.0 ± 5.1% larger). However, the differences between terrestrial and semi-aquatic species was small, only six species in our dataset are semi-aquatic, and a model with these three habitats is statistically inferior to one with two (AIC −405.8 versus −408.3).

For species with a mean adult female SVL of 800 mm, neonates are about 300 mm SVL in sea snakes versus 200 mm SVL in terrestrial snakes (figure 1). These figures correspond to a mass difference of approximately 10 g versus 35 g [21,43] (and unpublished data from R. Shine, 2023). Nonetheless, a few marine snakes (notably *Hydrophis schistosus*) have progeny almost as small as do terrestrial snakes of the same adult female size (figure 1). Part of the difference in offspring sizes between marine and non-marine snakes could be explained by the smaller litters of marine species. The broods of marine snakes are, on average, only 65.8% (SE = 7.1%, *p* < 0.0001, *n* = 18 marine species and 139 non-marine species with data on brood size) as large as the broods of non-marine snakes of the same female SVL. Even when controlling for brood size, however, marine species have longer neonates (by 27.4 ± 4.3%, *p* < 0.0001, model *R*^2^ = 0.889, *λ* = 0). The exclusion of *H. kingii* as an outlier did not affect any of our conclusions.

### 3.2. Experimental test of size-dependent risk of predation for small marine snakes

**Figure 2.**
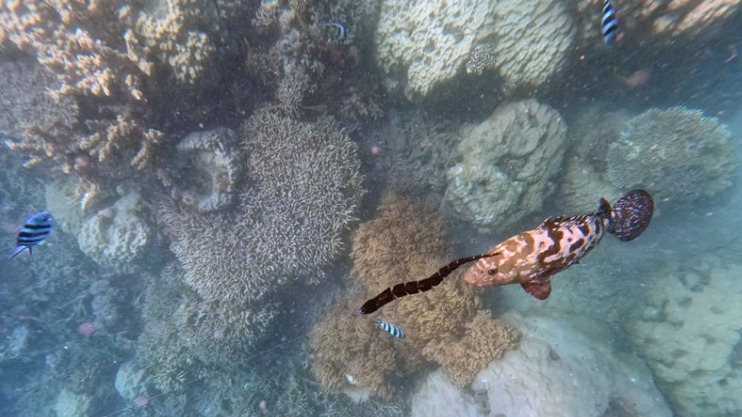
A camouflage grouper (*Epinephelus polyphekadion*) following a black snake-shaped lure, immediately prior to launching an attack. Photograph by Terri Shine.

A total of 47 trials resulted in 114 response records comprising 74 to small lures and 40 to large lures. In total we recorded 38 attacks and 76 follows (15 trials with at least one attack, 10 trials without attacks). Attacks to the lure were primarily by three large-bodied species: long toms *Tylosurus crocodilus* (11 attacks, 10 follows), camouflage groupers *Epinephelus polyphekadion* (11 attacks, 5 follows; figure 2), and coral trout *Plectropomus leopardus* (8 attacks, 17 follows). Smaller fish species generally followed rather than attacked the lures (blue trevally *Caranx melampygus N* = 1, 9; yellowtail emperor *Lethrinus atkinsoni N* = 3, 17; thumbprint emperor *L. harak N* = 4, 18).

**Figure 3.**
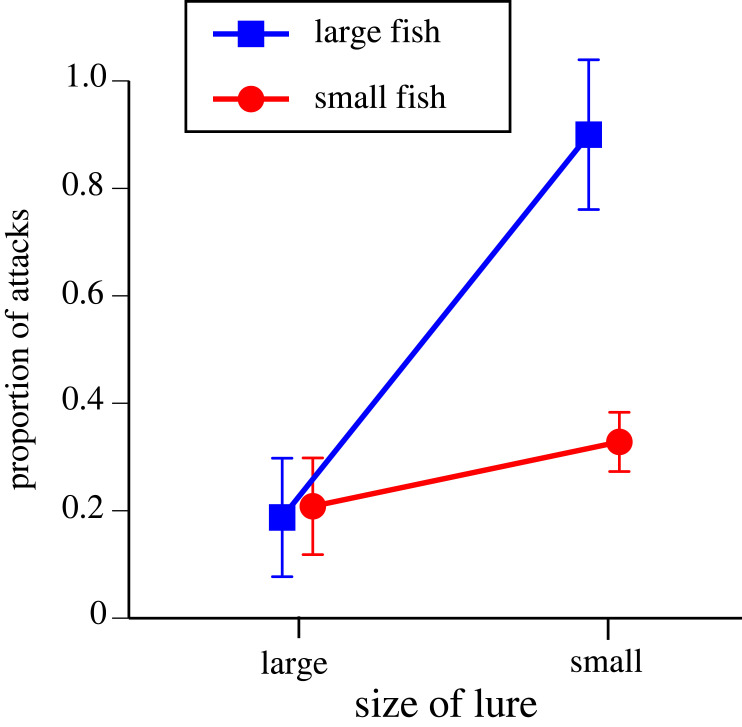
Rates of attack by predatory fishes on snake-shaped fibreglass lures in a coral-reef site in New Caledonia. Lures were of two sizes (300 mm and 200 mm long, shown as ‘large’ and ‘small’ in the figure). Sizes of fishes that attacked (seized) the lures also were classed as ‘large’ (greater than 600 mm long) and ‘small’ (less than 600 mm long). The Y-axis shows the number of cases in which a lure was attacked, as a proportion of all cases in which a fish either followed or attacked the lure (i.e. 0 = only follows, no attacks; 1 = all follows escalated to attacks).

The size of a lure did not significantly affect the number of follows (*F*_1,36_ = 0.82, *p* = 0.37) but smaller lures attracted more attacks (*F*_1,36_ = 5.47, *p* < 0.026). The two-factor analysis to explore the role of predator size relative to lure size generated a non-significant interaction term (*F*_1,97.73_ = 1.33, *p* = 0.25) but smaller lures were more likely to be attacked rather than followed (*F*_1,30.69_ = 5.87, *p* < 0.022) and larger fishes were more likely to attack rather than follow a lure (*F*_1,84.54_ = 5.98, *p* < 0.017). In summary, most attacks were directed to small lures by large fishes (figure 3).

## 4. Discussion

The invasion of marine habitats by snakes has been accompanied by an increase in relative offspring size, accompanied by a decrease in brood size (due, at least in part, to the increased size of offspring). That increase in offspring size plausibly reflects an evolutionary response to an increased susceptibility of neonatal snakes to predation. The vulnerability of small sea snakes is amplified by the abundance of large fast-moving predatory fish in coral-reef habitats, and the need for snakes to frequently leave the protection of substrate-associated shelter to ascend to the water surface to obtain oxygen. Many terrestrial habitats contain fewer large carnivores, and snakes can remain in sheltered sites where they are undetectable by avian predators for long periods of time. In keeping with those ideas, small snake-shaped lures pulled through the water at our study area in New Caledonia attracted frequent attacks by large fishes. Lures the size of neonatal sea snakes were less likely to be attacked than were otherwise-identical lures the size of neonatal terrestrial snakes.

Even in circumstances where sea snakes of all body sizes are equally likely to be attacked by predators, larger size may render a snake more capable of escaping and surviving the encounter. In two cases recorded near our study site, sea snakes were seized but then released by fishes too small to overpower the reptile (chocolate grouper *Cephalopholis boenak* attacking *Emydocephalus annulatus* – https://www.inaturalist.org/observations/116240077; stonefish *Synanceia verrucosa* attacking sea krait *Laticauda laticaudata* –https://www.inaturalist.org/observations/138403535). Hence, a larger size at birth may enhance survival rates of snakes even if smaller snakes are no more likely to be the target of attempted predation.

Predation is often suggested to be an important selective force on organismal traits, but direct empirical evidence of phenotype-dependent vulnerability is rare. That scarcity of evidence is especially true for vertebrates, for which ethical and logistical issues discourage experimental studies of predation in the field [44,45]. Demonstrations of differential predation relative to phenotype (colour) using clay models of snakes have been criticized on the grounds that disturbance to models may be due to curiosity or scavenging rather than predation [46]. In a few natural systems rates of predation may be high enough that observers can quantify selection on traits such as size and colour [23]. Ethical and logistical constraints are less stringent for studies on invertebrates with field and laboratory evidence of non-random predation on several insect species [47].

Vulnerability to predation is not the only possible selective advantage for the evolution of increased offspring size in marine snakes, but alternative ideas are less well-supported by evidence. For example, because marine snakes must swim rather than (or as well as) crawl, optimal offspring size might increase if larger body size enhances swimming ability more than crawling ability. Contrary to that assumption, however, swimming speed relative to crawling speed was higher for smaller sea kraits (Laticaudinae) than for larger conspecifics [48,49]. A second alternative is that larger size at birth may be favoured if the juveniles of marine snakes feed on larger prey items than do neonatal terrestrial snakes, and thus require larger trophic structures to process those prey items. By analogy, an increase in size at birth in insular populations of terrestrial snakes (relative to mainland taxa) has been attributed to gape-limitation combined with a lack of small prey on the islands [7,8,50]. Contrary to this idea, however, many sea snakes feed on small prey. For example, species of the genus *Emydocephalus* consume tiny fish eggs [51], and several *Hydrophis*-lineage species exhibit miniaturized heads and slender forebodies that enable them to penetrate the burrows of the small fishes upon which they prey [52]. Gape-limitation thus is not a general feature of sea snake biology. Another possible selective advantage of larger size at birth is to enhance success in competition for resources, with larger individuals excluding smaller conspecifics from high-quality habitats or resources; but agonistic interactions have never been reported for juvenile marine snakes [34]. Lastly, a snake's body size can affect its rates of heating and cooling in terrestrial species, but the high conductivity of water virtually eliminates such effects in marine taxa [53]. In summary, published data do not support the ideas that larger size at birth in marine snakes confers major advantages in thermal biology, swimming speed, intraspecific competition or in the ability to ingest prey items.

Nonetheless, our data do not provide definitive support for the hypothesis that size-selective predation has driven the evolution of larger size at birth in marine snakes. All we have done is show, for a single study system, that predation on marine snakes can be both intense and size-selective. To establish generality, studies on other marine systems are needed. Also, we need equivalent studies on terrestrial snakes to compare the intensity of size-selective predation between marine and terrestrial systems. Studies using immobile models in terrestrial systems have reported attack rates of 2% to 19% over the course of prolonged deployment (typically, several days [23]). These are orders of magnitude lower than recorded in our own study (38 attacks in 25 trials, each lasting only a few minutes), but comparison is weakened by the difference in stimuli used. Ideally, we need innovative methods that expose snakes (or snake-like models) of different sizes to terrestrial predators, to trigger attack by ambush-hunters as well as active-foraging predators. We predict that over the critical size range (200 to 300 mm length), rates of predation will be lower, and less sensitive to body size, in terrestrial systems than in marine systems.

Future work also could explore interspecific variation in offspring size within sea snakes, and within terrestrial elapids, to look for habitat correlates of life-history traits. For example, predation pressure likely is lower in shallow mud-flat habitats than in coral-reef sites, consistent with the small offspring of *Hydrophis schistosus* [54]. We might also expect pregnant female sea snakes to give birth in sites where large predatory fish are uncommon, or are less able to capture small snakes because of turbid water. In keeping with that prediction, females of some sea snake species actively select shallow-water habitats around the time of birth [54].

Our hypothesis that air-breathing by marine snakes imposes strong selection on minimum body size (and thus, offspring size) can be framed in a broader context. In some situations, an organism can meet all of its requirements without leaving a safe refuge—but if different resources are available in different places, the animal must shuttle between sites to access those resources. For example, a heliothermic lizard must forego its safe shelter to access a sun-exposed basking site, at the risk of increased vulnerability to predation [55,56]. Some species reduce that cost by basking within sheltered but warm sites [57]. Invading a novel habitat type can amplify the spatial disconnect between sites suitable for foraging and for other activities. For example, oviparous snakes in cooler parts of their range may need to migrate to open sun-exposed sites to obtain nesting conditions warm enough for embryonic development; and females undertaking those nesting migrations may be at risk from predation [58]. Similarly, the northward expansion of snake populations into central Canada as glaciers receded resulted in an increasing scarcity of overwintering sites deep enough to avoid lethally low midwinter temperatures [59]. As a result, garter snakes in these areas must seasonally migrate long distances between summer feeding ranges and winter dens, with high mortality as a result [60].

The invasion of the oceans by air-breathing vertebrates likewise imposed a spatial disconnect between feeding habitats and the location of breathable oxygen. The distance between safe refuges on the ocean floor and oxygen in the air above often is small, but the dangerous transit between substrate and water surface must be undertaken frequently. Adaptations that increase the duration of dives (e.g. cutaneous oxygen uptake, scale rugosity that affects boundary-layer gas exchange [31,61,62]) reduce but do not eliminate that risk. That mismatch between air-breathing and marine life may have imposed intense selection on traits such as locomotor ability and neonatal body size in sea snakes.

## Data Availability

Supplementary material is available online [63].
